# DNA vaccine protection against SARS-CoV-2 in rhesus macaques

**DOI:** 10.1126/science.abc6284

**Published:** 2020-05-20

**Authors:** Jingyou Yu, Lisa H. Tostanoski, Lauren Peter, Noe B. Mercado, Katherine McMahan, Shant H. Mahrokhian, Joseph P. Nkolola, Jinyan Liu, Zhenfeng Li, Abishek Chandrashekar, David R. Martinez, Carolin Loos, Caroline Atyeo, Stephanie Fischinger, John S. Burke, Matthew D. Slein, Yuezhou Chen, Adam Zuiani, Felipe J. N. Lelis, Meghan Travers, Shaghayegh Habibi, Laurent Pessaint, Alex Van Ry, Kelvin Blade, Renita Brown, Anthony Cook, Brad Finneyfrock, Alan Dodson, Elyse Teow, Jason Velasco, Roland Zahn, Frank Wegmann, Esther A. Bondzie, Gabriel Dagotto, Makda S. Gebre, Xuan He, Catherine Jacob-Dolan, Marinela Kirilova, Nicole Kordana, Zijin Lin, Lori F. Maxfield, Felix Nampanya, Ramya Nityanandam, John D. Ventura, Huahua Wan, Yongfei Cai, Bing Chen, Aaron G. Schmidt, Duane R. Wesemann, Ralph S. Baric, Galit Alter, Hanne Andersen, Mark G. Lewis, Dan H. Barouch

**Affiliations:** 1 Center for Virology and Vaccine Research, Beth Israel Deaconess Medical Center, Harvard Medical School, Boston, MA 02215, USA.; 2 Department of Epidemiology, University of North Carolina at Chapel Hill, Chapel Hill, NC 27599, USA.; 3 Ragon Institute of MGH, MIT, and Harvard, Cambridge, MA 02139, USA.; 4 Brigham and Women’s Hospital, Harvard Medical School, Boston, MA 02115, USA.; 5 Bioqual, Rockville, MD 20852, USA.; 6 Janssen Vaccines & Prevention BV, Leiden, Netherlands.; 7 Children’s Hospital, Boston, MA 02115, USA.; 8 Massachusetts Consortium on Pathogen Readiness, Boston, MA 02215, USA.

## Abstract

The global coronavirus disease 2019 (COVID-19) pandemic caused by severe acute respiratory syndrome coronavirus 2 (SARS-CoV-2) has made the development of a vaccine a top biomedical priority. In this study, we developed a series of DNA vaccine candidates expressing different forms of the SARS-CoV-2 spike (S) protein and evaluated them in 35 rhesus macaques. Vaccinated animals developed humoral and cellular immune responses, including neutralizing antibody titers at levels comparable to those found in convalescent humans and macaques infected with SARS-CoV-2. After vaccination, all animals were challenged with SARS-CoV-2, and the vaccine encoding the full-length S protein resulted in >3.1 and >3.7 log_10_ reductions in median viral loads in bronchoalveolar lavage and nasal mucosa, respectively, as compared with viral loads in sham controls. Vaccine-elicited neutralizing antibody titers correlated with protective efficacy, suggesting an immune correlate of protection. These data demonstrate vaccine protection against SARS-CoV-2 in nonhuman primates.

## Prototype DNA vaccines for SARS-CoV-2

The development of a vaccine to protect against severe acute respiratory syndrome coronavirus 2 (SARS-CoV-2) is an urgent biomedical need. Yu *et al.* designed a series of prototype DNA vaccines against the SARS-CoV-2 spike protein, which is used by the virus to bind and invade human cells. Analysis of the vaccine candidates in rhesus macaques showed that animals developed protective humoral and cellular immune responses when challenged with the virus. Neutralizing antibody titers were also observed at levels similar to those seen in humans who have recovered from SARS-CoV-2 infection.

*Science*, this issue p. 806

## Teaser

Successful protection against SARS-CoV-2 in rhesus macaques by means of a prototype DNA vaccine is described.

The coronavirus disease 2019 (COVID-19) pandemic has made the development of a safe, effective, and deployable vaccine to protect against infection with severe acute respiratory syndrome coronavirus 2 (SARS-CoV-2) a critical global priority (*1*–*8*). Our current understanding of immune correlates of protection against SARS-CoV-2 is limited but will be essential to enable the development of SARS-CoV-2 vaccines and other immunotherapeutic interventions. To facilitate the preclinical evaluation of vaccine candidates, we recently developed a rhesus macaque model of SARS-CoV-2 infection (*9*). In the present study, we constructed a set of prototype DNA vaccines expressing various forms of the SARS-CoV-2 spike (S) protein and assessed their immunogenicity and protective efficacy against SARS-CoV-2 viral challenge in rhesus macaques.

## Construction and immunogenicity of DNA vaccine candidates

**Fig. 1:**
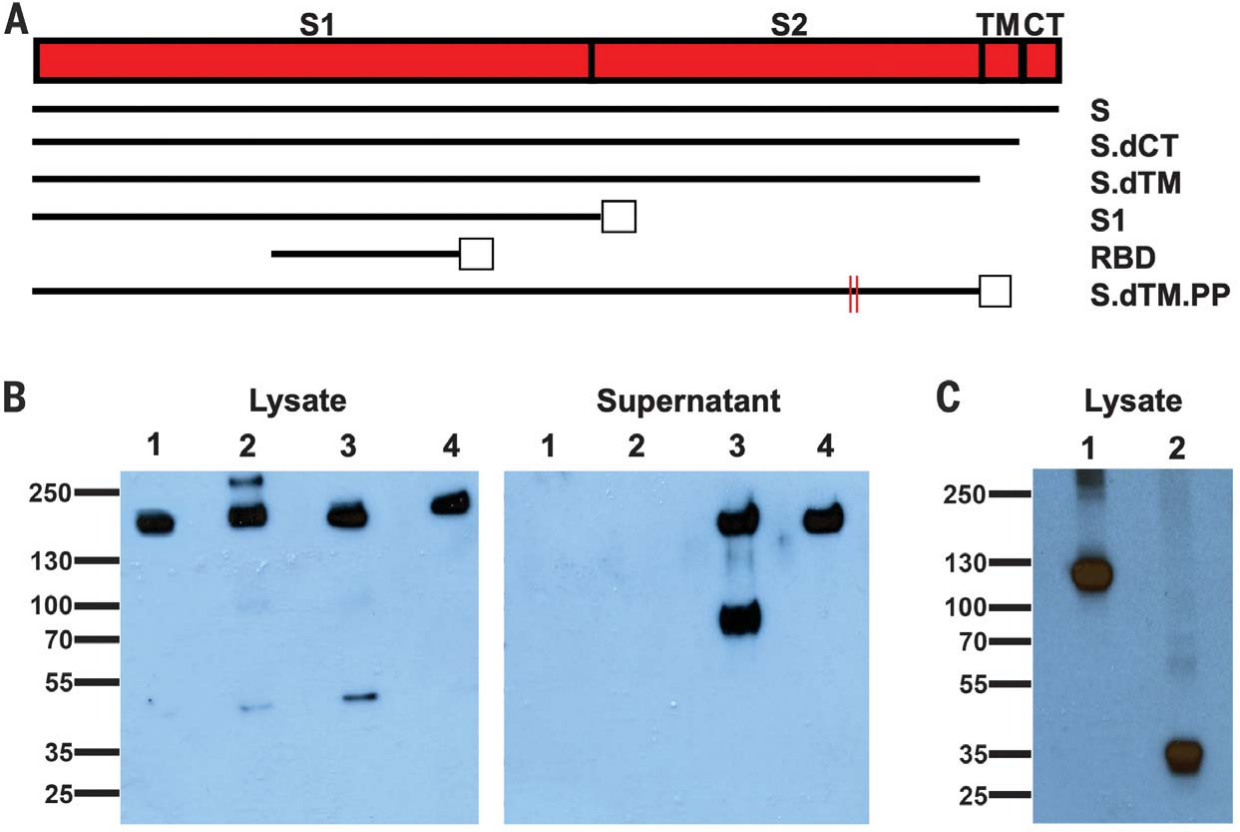
Construction of candidate DNA vaccines against SARS-CoV-2. (**A**) Six DNA vaccines were produced expressing different SARS-CoV-2 spike (S) variants: (i) full length (S), (ii) deletion of the cytoplasmic tail (S.dCT), (iii) deletion of the transmembrane (TM) domain and cytoplasmic tail (CT) reflecting the soluble ectodomain (S.dTM), (iv) S1 domain with a foldon trimerization tag (S1), (v) receptor-binding domain with a foldon trimerization tag (RBD), and (vi) prefusion-stabilized soluble ectodomain with deletion of the furin cleavage site, two proline mutations, and a foldon trimerization tag (S.dTM.PP). Open squares depict foldon trimerization tags; red lines depict proline mutations. (**B**) Western blot analyses for expression from DNA vaccines encoding S (lane 1), S.dCT (lane 2), S.dTM (lane 3), and S.dTM.PP (lane 4) in cell lysates and culture supernatants using an anti-SARS polyclonal antibody (BEI Resources). (**C**) Western blot analyses for expression from DNA vaccines encoding S1 (lane 1) and RBD (lane 2) in cell lysates using an anti–SARS-CoV-2 RBD polyclonal antibody (Sino Biological).

We produced a series of prototype DNA vaccines expressing six variants of the SARS-CoV-2 S protein: (i) full length (S), (ii) deletion of the cytoplasmic tail (S.dCT) (*10*), (iii) deletion of the transmembrane domain and cytoplasmic tail reflecting the soluble ectodomain (S.dTM) (*10*), (iv) S1 domain with a foldon trimerization tag (S1), (v) receptor-binding domain with a foldon trimerization tag (RBD), and (vi) a prefusion-stabilized soluble ectodomain with deletion of the furin cleavage site, two proline mutations, and a foldon trimerization tag (S.dTM.PP) (*11*–*13*) (Fig. 1A). Western blot analyses confirmed expression in cell lysates for all constructs and in culture supernatants for the soluble S.dTM and S.dTM.PP constructs (Fig. 1, B and C). Proteolytic cleavage of the secreted protein was noted for S.dTM but not S.dTM.PP, presumably as a result of mutation of the furin cleavage site in S.dTM.PP.

**Fig. 2:**
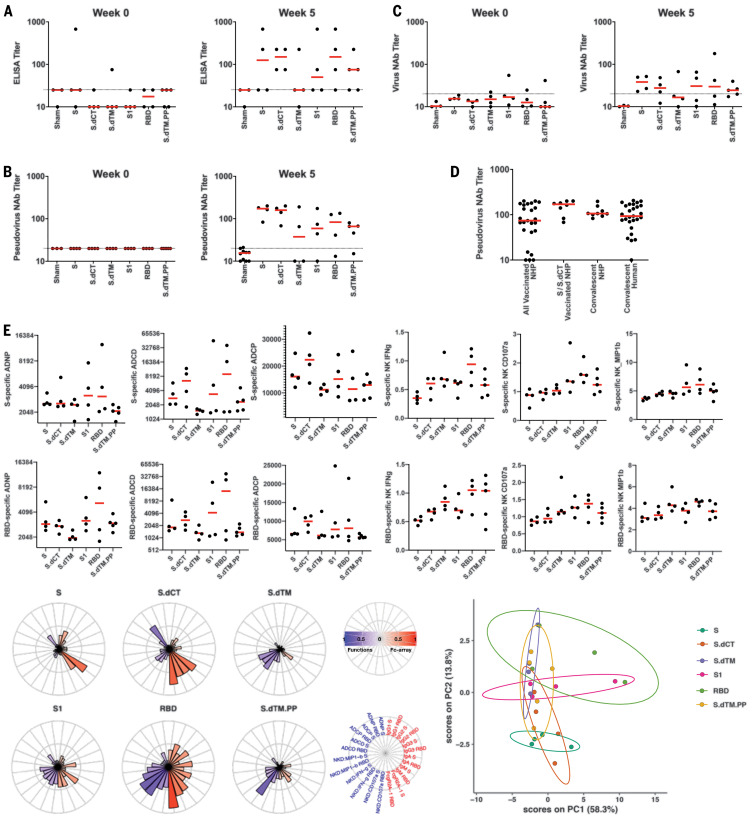
Humoral immune responses in vaccinated rhesus macaques. (**A** to **C**) Humoral immune responses were assessed after immunization by (A) binding antibody ELISA, (B) pseudovirus neutralization assays, and (C) live virus neutralization assays. (**D**) Comparison of pseudovirus neutralization titers in vaccinated macaques (all animals as well as the S and S.dCT groups), a cohort of 9 convalescent macaques, and a cohort of 27 convalescent humans from Boston, United States, who had recovered from SARS-CoV-2 infection. NHP, nonhuman primates. (**E**) S- and RBD-specific antibody-dependent neutrophil phagocytosis (ADNP), antibody-dependent complement deposition (ADCD), antibody-dependent monocyte cellular phagocytosis (ADCP), and antibody-dependent NK cell activation (IFN-γ secretion, CD107a degranulation, and MIP-1β expression) are shown. Radar plots show the distribution of antibody features across the vaccine groups. The size and color intensity of the wedges indicate the median of the feature for the corresponding group (blue depicts antibody functions; red depicts antibody isotype, subclass, and FcγR binding). The principal components analysis (PCA) plot shows the multivariate antibody profiles across groups. Each dot represents an animal, the color of the dot denotes the group, and the ellipses show the distribution of the groups as 70% confidence levels assuming a multivariate normal distribution. In the dot plots above, red bars reflect median responses, and dotted lines reflect assay limits of quantitation.

We immunized 35 adult rhesus macaques (6 to 12 years old) with DNA vaccines in the following groups: S (*N* = 4), S.dCT (*N* = 4), S.dTM (*N* = 4), S1 (*N* = 4), RBD (*N* = 4), S.dTM.PP (*N* = 5), and sham controls (*N* = 10). Animals received 5-mg DNA vaccines by the intramuscular route without adjuvant at weeks 0 and 3. After the boost immunization at week 5, we observed S-specific binding antibodies by enzyme-linked immunosorbent assay (ELISA) (Fig. 2A) and neutralizing antibodies (NAbs) by both a pseudovirus neutralization assay (*10*) (Fig. 2B) and a live virus neutralization assay (*14*, *15*) (Fig. 2C). As determined by ELISA, two animals had binding antibodies at baseline, which might reflect cross-reactivity of other natural primate coronaviruses. NAb titers measured by the pseudovirus neutralization assay correlated with NAb titers measured by the live virus neutralization assay (*P* < 0.0001, *R* = 0.8052, two-sided Spearman rank-correlation test; fig. S1). Moreover, NAb titers in the vaccinated macaques (median titer = 74; median titer in the S and S.dCT groups = 170) were comparable in magnitude to NAb titers in a cohort of 9 convalescent macaques (median titer = 106) and a cohort of 27 convalescent humans (median titer = 93) who had recovered from SARS-CoV-2 infection (Fig. 2D).

S-specific and RBD-specific antibodies in the vaccinated macaques included diverse subclasses and effector functions, including antibody-dependent neutrophil phagocytosis (ADNP), antibody-dependent complement deposition (ADCD), antibody-dependent monocyte cellular phagocytosis (ADCP), and antibody-dependent natural killer (NK) cell activation [interferon-γ (IFN-γ) secretion, CD107a degranulation, and MIP-1β expression] (*16*) (Fig. 2E). A trend toward higher ADCD responses was observed in the S and S.dCT groups, whereas higher NK cell activation was observed in the RBD and S.dTM.PP groups. A principal components analysis of the functional and biophysical antibody features showed overlap of the different vaccine groups, with more distinct profiles in the S and RBD groups (Fig. 2E).

**Fig. 3:**
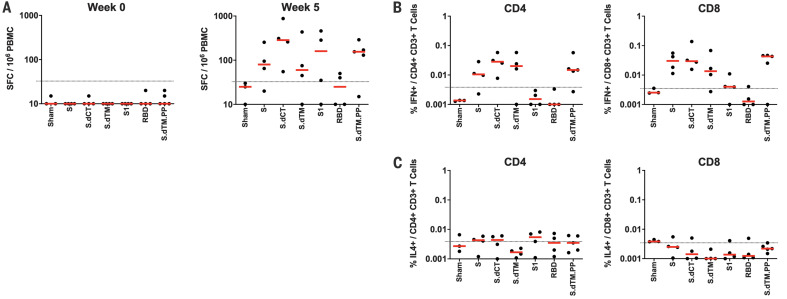
Cellular immune responses in vaccinated rhesus macaques. At week 5 after immunization, cellular immune responses were assessed by (**A**) IFN-γ ELISPOT assays and (**B**) IFN-γ+ and (**C**) IL-4+ intracellular cytokine staining assays for CD4+ and CD8+ T cells in response to pooled S peptides. Red bars reflect median responses; dotted lines reflect assay limits of quantitation.

We also observed cellular immune responses to pooled S peptides in most vaccinated animals by IFN-γ enzyme-linked immunosorbent spot (ELISPOT) assays at week 5 (Fig. 3A). Intracellular cytokine staining assays at week 5 demonstrated induction of S-specific IFN-γ+ CD4+ and CD8+ T cell responses, with lower responses induced by the shorter S1 and RBD immunogens (Fig. 3B). S-specific IL-4+ CD4+ and CD8+ T cell responses were marginal (Fig. 3C), suggesting induction of T helper 1 (T_H_1)–biased cellular immune responses.

## Protective efficacy against SARS-CoV-2 challenge

At week 6, which was 3 weeks after the boost immunization, all animals were challenged with 1.2 × 10^8^ virus particles (VPs) [1.1 × 10^4^ plaque-forming units (PFUs)] of SARS-CoV-2, administered as 1 ml by the intranasal route and 1 ml by the intratracheal route. After challenge, we assessed viral RNA levels by reverse transcription polymerase chain reaction (*17*) in bronchoalveolar lavage (BAL) and nasal swabs (NS). Viral RNA was negative in plasma, and animals exhibited only mild clinical symptoms. High levels of viral RNA were observed in the sham controls, with a median peak of 6.46 (range = 4.81 to 7.99) log_10_ RNA copies/ml in BAL and a median peak of 6.82 (range = 5.96 to 7.96) log_10_ RNA copies/swab in NS (fig. S2). Lower levels of viral RNA were observed in the vaccine groups (figs. S3 and S4), including 1.92 and 2.16 log_10_ reductions of median peak viral RNA in BAL and NS, respectively, in S-vaccinated animals compared with sham controls (*P* = 0.02 and 0.04, two-sided Mann-Whitney tests) (fig. S5). Viral RNA assays were confirmed by PFU assays, which similarly showed lower infectious virus titers in S-vaccinated animals compared with sham controls (*P* = 0.04, two-sided Mann-Whitney test) (fig. S5).

**Fig. 4:**
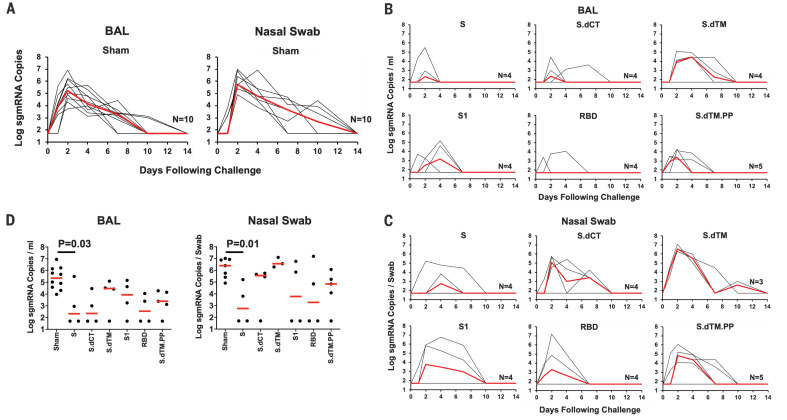
Viral loads in rhesus macaques challenged with SARS-CoV-2 virus. Rhesus macaques were challenged via the intranasal and intratracheal routes with 1.2 × 10^8^ VPs (1.1 × 10^4^ PFUs) of SARS-CoV-2. (**A**) Log_10_ sgmRNA copies per milliliter or copies per swab (limit 50 copies) were assessed in bronchoalveolar lavage (BAL) and nasal swabs (NS) in sham controls at multiple time points after challenge. (**B**) Log_10_ sgmRNA copies per milliliter in BAL and (**C**) log_10_ sgmRNA copies per swab in NS in vaccinated animals at multiple time points after challenge. (**D**) Summary of peak viral loads in BAL and NS after challenge. Peak viral loads occurred variably on days 1 to 4 after challenge. Red lines reflect median viral loads. *P* values indicate two-sided Mann-Whitney tests.

We speculated that a substantial fraction of viral RNA in BAL and NS after challenge represented input challenge virus. Therefore, we also assessed levels of subgenomic mRNA (sgmRNA), which are believed to reflect viral replication cellular intermediates that are not packaged into virions, and thus putative replicating virus in cells (*18*). High levels of sgmRNA were observed in the sham controls (Fig. 4A) with a median peak of 5.35 (range = 3.97 to 6.95) log_10_ sgmRNA copies/ml in BAL and 6.40 (range = 4.91 to 7.01) log_10_ sgmRNA copies per swab in NS. Peak viral loads occurred variably on days 1 to 4 after challenge. Markedly lower levels of sgmRNA were observed in the vaccine groups (Fig. 4, B and C), including >3.1 and >3.7 log_10_ decreases of median peak sgmRNA in BAL and NS, respectively, in S-vaccinated animals compared with sham controls (*P* = 0.03 and 0.01, two-sided Mann-Whitney tests) (Fig. 4D). Reduced levels of sgmRNA were also observed in other vaccine groups, including S.dCT, S1, RBD, and S.dTM.PP, although minimal to no protection was seen in the S.dTM group, confirming the importance of prefusion ectodomain stabilization, as reported previously (*13*). Protection was generally more robust in BAL compared with NS, particularly for the less immunogenic constructs. A total of 8 of 25 vaccinated animals exhibited no detectable sgmRNA in BAL and NS at any time point after challenge.

## Immune correlates of vaccine-induced protection

**Fig. 5:**
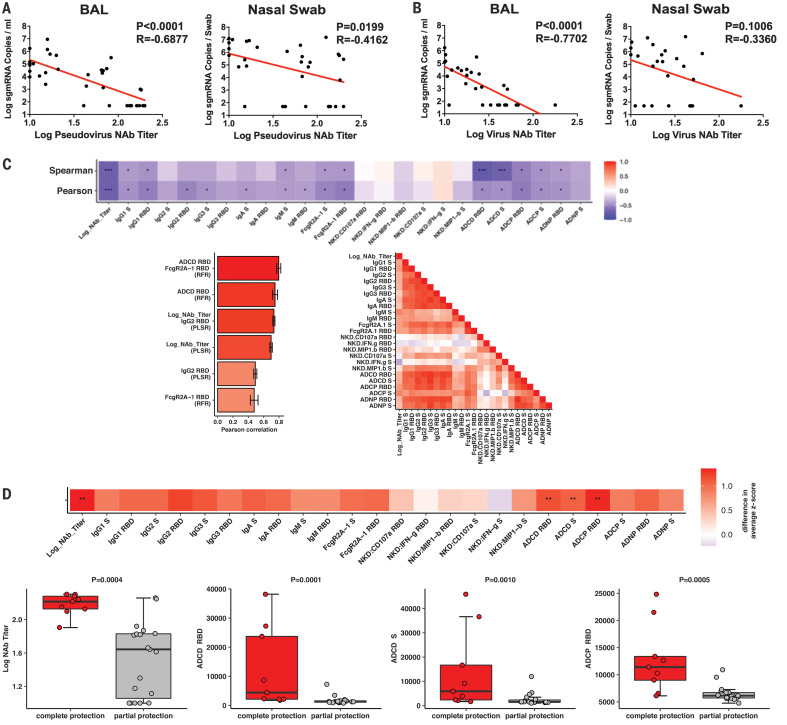
Immune correlates of protection. (**A** and **B**) Correlations of (A) pseudovirus NAb titers and (B) live NAb titers before challenge with log peak sgmRNA copies per milliliter in BAL or log peak sgmRNA copies per swab in nasal swabs after challenge. Red lines reflect the best-fit relationship between these variables. *P* and *R* values reflect two-sided Spearman rank-correlation tests. (**C**) The heatmap (top) shows the Spearman and Pearson correlations between antibody features and log_10_ peak sgmRNA copies per milliliter in BAL (*q < 0.05, **q < 0.01, ***q < 0.001 with Benjamini-Hochberg correction for multiple testing). The bar graph (bottom left) shows the rank of the Pearson correlation between cross-validated model predictions and data using the most predictive combination or individual antibody features for partial least squares regression (PLSR) and random forest regression (RFR). Error bars indicate SEs. The correlation heatmap (bottom right) represents pairwise Pearson correlations between features across all animals. (**D**) The heatmap (top) shows the difference in the means of the *z*-scored features between the completely protected and partially protected animals (**q < 0.01 with Benjamini-Hochberg correction for multiple testing). The dot plots show differences in log_10_ NAb titers, RBD-specific ADCD responses, S-specific ADCD responses, and RBD-specific ADCP responses between the completely protected and partially protected animals. *P* values indicate two-sided Mann-Whitney tests.

The variability in protective efficacy in this study facilitated an analysis of immune correlates of protection. The log_10_ pseudovirus NAb titer at week 5 inversely correlated with peak log_10_ sgmRNA in both BAL (*P* < 0.0001, *R* = −0.6877, two-sided Spearman rank-correlation test) and NS (*P* = 0.0199, *R* = −0.4162) (Fig. 5A). Similarly, the log_10_ live virus NAb titer at week 5 inversely correlated with peak log_10_ sgmRNA levels in both BAL (*P* < 0.0001, *R* = −0.7702) and NS (*P* = 0.1006, *R* = −0.3360) (Fig. 5B). These data suggest that vaccine-elicited serum NAb titers may be immune correlates of protection against SARS-CoV-2 challenge. We speculate that correlations were more robust with viral loads in BAL compared with viral loads in NS, due to intrinsic variability of collecting swabs. The log_10_ ELISA titer at week 5 also inversely correlated with peak log_10_ sgmRNA levels in BAL (*P* = 0.0041, *R* = −0.4733) (fig. S6). Vaccine-elicited ELISPOT responses (fig. S7), CD4+ intracellular cytokine staining (ICS) responses (fig. S8), and CD8+ ICS responses (fig. S9) did not correlate with protection.

We next explored the potential contribution of other antibody effector functions to immune correlates of protection. In addition to NAb titers, S- and RBD-specific ADCD responses inversely correlated with peak log_10_ sgmRNA levels in BAL (Fig. 5C, top). Two orthogonal unbiased machine learning approaches were then used to define minimal combined correlates of protection. A nonlinear random forest regression analysis and a linear partial least squares regression analysis showed that using two features improved the correlations with protection, such as RBD-specific FcγR2a-1 binding with ADCD responses or NAb titers with RBD-specific IgG2 responses (Fig. 5C, bottom left). Moreover, NAb titers correlated with most antibody effector functions, except for antibody-mediated NK cell activation (Fig. 5C, bottom right). Taken together, these data suggest that NAbs have a primary role in protecting against SARS-CoV-2, supported by certain innate immune effector functions such as ADCD.

Finally, we compared antibody parameters in vaccinated animals that were completely protected (defined as no detectable sgmRNA after challenge) with those in vaccinated animals that were partially protected (defined as detectable sgmRNA after challenge). Log_10_ NAb titers (*P* = 0.0004, two-sided Mann-Whitney test), RBD-specific ADCD responses (*P* = 0.0001), S-specific RBD responses (*P* = 0.0010), and RBD-specific ADCP responses (*P* = 0.0005) were higher in completely protected animals than in partially protected animals (Fig. 5D).

## Anamnestic immune responses after challenge

All animals exhibited anamnestic humoral and cellular immune responses after challenge, including increased ELISA titers (fig. S10), pseudovirus NAb titers (fig. S11), live virus NAb titers (fig. S12), and IFN-γ ELISPOT responses (fig. S13) on day 14 after challenge. These data suggest that vaccine protection was probably not sterilizing (including in the 8 of 25 animals that had no detectable sgmRNA in BAL and NS at any time point after challenge) but rather was likely mediated by rapid virologic control after challenge.

## Discussion

A safe and effective SARS-CoV-2 vaccine may be required to end the global COVID-19 pandemic. Several vaccine candidates have initiated clinical testing, and many others are in preclinical development (*19*, *20*). However, very little is currently known about immune correlates of protection and protective efficacy of candidate SARS-CoV-2 vaccines in animal models. In this study, we generated a series of prototype DNA vaccines expressing various S immunogens and assessed protective efficacy against intranasal and intratracheal SARS-CoV-2 challenge in rhesus macaques. We demonstrated vaccine protection with substantial >3.1 and >3.7 log_10_ reductions in median viral loads in BAL and NS, respectively, in S-immunized animals compared with sham controls. Protection was likely not sterilizing but instead appeared to be mediated by rapid immunologic control after challenge.

Our data extend the findings of previous studies on SARS and Middle East respiratory syndrome (MERS) vaccine protection in mice, ferrets, and macaques (*10*, *21*–*24*). Phase 1 clinical studies for SARS and MERS vaccine candidates have also been conducted (*25*), but these vaccines have not been tested for efficacy in humans. Our data suggest that vaccine protection against SARS-CoV-2 in macaques is feasible. We observed a marked reduction of viral replication in both the upper respiratory tract and the lower respiratory tract with the optimal vaccines. By contrast, the less immunogenic vaccines, such as S.dTM, showed partial protection in BAL but essentially no protection in NS. These data suggest that it may be easier to protect against lower respiratory tract disease than against upper respiratory tract disease. In the present study, optimal protection was achieved with the full-length S immunogen in both the upper and lower respiratory tracts, and reduced protection was observed with soluble constructs and smaller fragments. Our study did not address the question of whether emerging mutations in the SARS-CoV-2 S sequence mediate escape from NAb responses induced by immunogens designed from the Wuhan/WIV04/2019 sequence.

Further research will need to address the durability of protective immunity and the optimal platforms for a SARS-CoV-2 vaccine for humans (*26*). Although our data are restricted to DNA vaccines, our findings may be generalizable to other gene-based vaccines as well, including RNA vaccines and recombinant vector-based vaccines. Additional research should also evaluate vaccine immunogenicity and protective efficacy in older animals. Future studies should also address the question of enhanced respiratory disease, which may result from antibody-dependent enhancement (*27*–*29*). Although our study was not designed to examine safety issues, it is worth noting that the DNA vaccines induced T_H_1 rather than T_H_2 responses, and we did not observe enhanced clinical disease even with the suboptimal vaccine constructs that failed to protect against infection.

We identified serum NAb titers, as measured by two independent assays (pseudovirus neutralization and live virus neutralization), as a significant correlate of protection against disease of both the lower and upper respiratory tracts. It is likely that protection in both anatomic compartments will be necessary for pandemic control, although protection in the upper respiratory tract may be more difficult to achieve. If this NAb correlate proves generalizable across multiple vaccine studies in both nonhuman primates and humans, then this parameter would be a simple and useful benchmark for clinical development of SARS-CoV-2 vaccines. Innate immune effector functions such as ADCD may also contribute to protective efficacy. In summary, we demonstrate effective vaccine protection against SARS-CoV-2 in rhesus macaques and define NAb titers as an immune correlate of protection, which will accelerate the development of SARS-CoV-2 vaccines for humans.

## Supplementary Materials

science.sciencemag.org/content/369/6505/806/suppl/DC1 Materials and Methods Figs. S1 to S13 References

## Appendix

View/request a protocol for this paper from *Bio-protocol*.
